# Nanotechnology and Drug Delivery: An Update in Oncology

**DOI:** 10.3390/pharmaceutics3020171

**Published:** 2011-04-14

**Authors:** Tait Jones, Nabil Saba

**Affiliations:** 1 Department of Medicine, Emory University, 80 Jesse Hill Dr., Atlanta, GA 30303, USA; E-Mail: ttjones@emory.edu; 2 Winship Cancer Institute, Department of Medicine, Emory University, 1365 Clifton Rd, Atlanta, GA 30322, USA

**Keywords:** drug delivery systems, nanotechnology, cancer, passive targeting, active targeting

## Abstract

The field of nanotechnology has exploded in recent years with diverse arrays of applications. Cancer therapeutics have recently seen benefit from nanotechnology with the approval of some early nanoscale drug delivery systems. A diversity of novel delivery systems are currently under investigation and an array of newly developed, customized particles have reached clinical application. Drug delivery systems have traditionally relied on passive targeting via increased vascular permeability of malignant tissue, known as the enhanced permeability and retention effect (EPR). More recently, there has been an increased use of active targeting by incorporating cell specific ligands such as monoclonal antibodies, lectins, and growth factor receptors. This customizable approach has raised the possibility of drug delivery systems capable of multiple, simultaneous functions, including applications in diagnostics, imaging, and therapy which is paving the way to improved early detection methods, more effective therapy, and better survivorship for cancer patients.

## 1. Introduction

Nanotechnology is the science of synthesis, manipulation, and utilization of custom built molecular machines, known as nanoparticles. Strictly defined, nanoparticles are molecules which are less than 100 nm in size, however, the term has been used more loosely to describe particles up to 500 nm [1]. Nanoparticles have been designed for applications in the fields of biochemistry and medicine, allowing investigators and clinicians to exploit a large number of these self-assembling, biocompatible products. The increase in number of drug delivery platforms made possible by these technologies is leading to a paradigm shift in a number of medical applications, particularly in the fields of oncology and pharmacokinetics.

Cancer is the second most common cause of mortality in the United States [2]. Although advances in early detection, newer formulations of chemotherapy, and the use of combined modality therapy have significantly improved survival in a number of tumors, there has been little change in overall cancer mortality. Treatment of patients with a large disease burden remains a challenge. This is heightened by the lack of early detection methods for a number of malignancies. In patients with significant tumor burden, systemic toxicity from conventional chemotherapy may limit drug delivery and lead to treatment failure. Conventional chemotherapeutic agents have generally been low molecular weight cytotoxins, which have a large volume of distribution and low plasma half-lives. A large number of these agents are lipid soluble, as they need to transverse the cell membrane, and are formulated with solvents that may have a significant toxicity profile themselves. There has been significant interest in developing drug delivery systems (DDS) incorporating biodegradable materials which would be capable of incorporating lipid soluble agents, increase the fraction of drug delivered to target tissues, and minimize exposure of healthy tissue to chemotherapy and toxic metabolites. DDS incorporating nanoparticles may be a future answer to some of these challenges.

As new drug delivery platforms have been identified, a means of classifying these particles by their method of reaching targeted tissue has also been developed. This has led to the distinction between actively and passively targeted DDS. Passively targeted DDS chiefly rely on the discrepancy in vascular permeability between healthy and malignant tissue. Nanoparticle based chemotherapeutic agents do not reach healthy tissues as they are too large to pass through the fenestrations of normal vasculature. As a result of disorganized angiogenesis, capillaries in malignant tissue are significantly larger and “leaky,” allowing access for DDS and delivery of chemotherapy to target tissues in tumors. Furthermore, the absence of normal lymphatic channels in tumor cells impairs drug clearance from these tissues and enhances the availability of chemotherapy for cell kill. The phenomenon of increased permeability of malignant tissue is known as the enhanced permeability and retention (EPR) effect [3-5]. The incorporation of chemotherapy into DDS is not without a downside, however. The use of nanoparticle constructs enhances the antigenicity of the DDS and makes it susceptible to opsonization and uptake by the reticuloendothelial system (RES). Polyethylene glycol (PEG) has frequently been incorporated into DDS to impair this process and increase bioavailability [6]. The PEG polymer creates a protective halo around the nanoparticle, preventing the attachment of circulating antibodies and impairing its clearance by phagocytic cells [7]. To address this issue, there has been significant attention in developing DDS that incorporate active targeting. A number of cellular targets such as growth factor receptor analogues, monoclonal antibodies, and other ligands are being studied as a means to facilitate direct delivery. Ligands covalently bound to nanoparticles bind directly to tumor cells, improving local drug concentration and facilitating endocytosis of the DDS.

## 2. Drug Delivery Systems

### 2.1. Liposomal formulations

**Figure 1.**
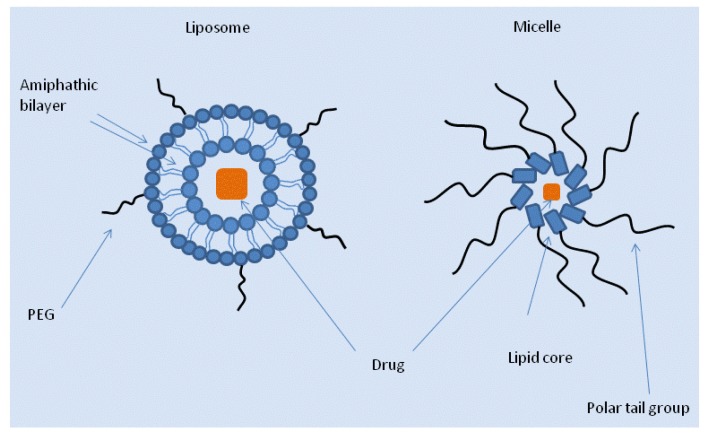
Diagrammatic Representation of Drug Delivery Systems (DDS).

Liposomal formulations were one of the first fusions of conventional chemotherapeutic drugs with nanoparticle-based DDS in a nanoformulation. Liposomes are constructs of molecules containing both hydrophobic and hydrophilic moieties, analogous to the phospholipids which compose the cell plasmalemma (Figure 1). They spontaneously self-assemble to create a spherical bilayer, generally with a hydrophilic exterior and hydrophobic interior. Since they produce a privileged internal environment and increase the diameter of the drug complex, they have been utilized as carrier complexes for drug delivery. Further, by manipulating the physical properties of the individual components, various liposomes of different sizes and physical properties can be constructed. For example, liposomes with hydrophobic or hydrophilic interiors can be created to facilitate delivery of various drugs. The earliest successful use of liposomes in oncology was applied to the delivery of anthracyclines, such as doxorubicin and daunorubucin. These drugs are thought to cause cytotoxicity by inhibition of nucleic acid synthesis and have been utilized in combination with other chemotherapy in a wide range of solid and liquid tumors. They were modified with the intent to decrease cardiotoxicity, a dose limiting side effect. In approximately 25% of patients who receive a lifetime dose of 500 mg/m^2^, doxorubicin causes sufficient damage to the heart to induce congestive heart failure [8-10]. Anthracycline induced heart failure is not always immediately apparent, and may manifest years after therapy is completed. Further, therapy with free doxorubicin may be severely limited by myelosuppression, stomatitis, and other side effects. The fusion of liposomes and anthracyclines produced a DDS capable of delivering a safer and more effective therapy.

In comparison to free drug, initial formulations of liposomal doxorubicin demonstrated superiority in early clinical trials [11-16]. Conventional lipsomal doxorbucin demonstrated an increased plasma concentration over time with reduced myelotoxicity in phase 1 trials. This improvement in bioavailability was thought to be due to protection from metabolism by the liposome and the higher doses tolerated by patients given the liposomal formulation. Rahman *et al* noted reduced urinary excretion of metabolites when using liposomal doxorubicin compared to free drug, suggesting that liposomal formulations may also reduce patient exposure to toxic metabolites [17]. Further, a reduction in minor adverse reactions, such as stomatitis, alopecia, venous sclerosis, nausea and emesis was noted across a variety of studies. Animal models have also demonstrated reduced cardiotoxicity with liposomal enhanced doxorubicin (LED) compared to free drug [18-22]. Doxil, a PEGylated formulation approved for clinical use in several malignancies, has a plasma half life of approximately 45 hours, compared to a plasma half-life of around 5 minutes for free doxorubicin and a significantly reduced volume of distribution compared to free drug [23].

The liposomal formulation incorporating PEG seems to reduce uptake by the RES and therefore increases the plasma concentrations of the drug. Unfortunately, PEGylation also increases accumulation of the drug in skin tissue, producing the side effect of hand-foot syndrome, also known as palmar-plantar erythrodysesthesia, a limiting factor for the novel formulation [24]. This side effect highlights the weakness of passively targeted drug delivery, as non-specific uptake of drug produces cell death away from target sites. This further emphasizes the need for the development of DDS utilizing active targeting systems. The superiority of liposomal anthracycline DDS to free drug has been well established and several liposomal formulations are approved for clinical use. A number of liposomal formulations incorporating other chemotherapeutic agents are under investigation in clinical trials (Table 1).

### 2.2. Micelles

Like liposomes, micelles are a form of lipid based DDS, comprised of a solid globule of amphipathic molecules with a polar “head” and hydrophobic “tail,” which form a hydrophobic core used to deliver hydrophobic chemotherapeutic agents. Like some liposomes, micelles reduce the risk of embolization that occurs with administration of hydrophobic drugs by stabilizing the drug and preventing aggregation of drug complexes in the blood [25,26]. When the individual components of the micelle incorporate certain polar molecules, such as PEG or polyethylene oxide (PEO), in the polymer, the micelle becomes intrinsically resistant to opsonization and uptake by the RES [27]. Drug may be incorporated into micelles either by agitation or by covalent attachment of drug to the individual polymer. Several therapeutics have been developed with a micelle based DDS and are under investigation in clinical trials, including paclitaxel, cisplatin, and doxorubicin based formulations [28,29]. One particularly interesting application of micelles has been their use in combination with ultrasound. Micelles may be disrupted by ultrasound, releasing the contents of the core. Thus, using ultrasound, micelles may be activated at target tissues, triggering release of drug and more specifically timed drug delivery [30]. Some of the disadvantages of micelles compared to liposomes include their diminished flexibility in accommodating both hydrophilic and hydrophobic molecules at their core compared to liposomal formulations as well as their relative instability due to smaller size, leading to quicker release of encapsulated drug [23].

### 2.3. Protein and polymer based formulations

Like liposomes, plasma proteins accumulate in tumor cells at higher concentrations than healthy cells. Albumin based drug formulations are analogous to liposomal DDS in that they have been designed to exploit the EPR effect to achieve a higher drug concentration in tumor cells. Albumin, a normal serum protein, allows efficient delivery of bound molecules directly to target cells, is capable of binding a large number of hydrophobic molecules, and is naturally endocytosed by cells. Further, some tumors upregulate the expression of albumin binding proteins for unclear reasons. Conjugation of chemotherapy to albumin or albumin-based polymers protects healthy tissue from free drug and metabolites, increases the plasma half-life, and improves drug delivery. Paclitaxel, a drug which induces cytotoxicity by derangement of the cell's cytoskeleton, has been conjugated to human albumin to produce the drug Abraxane, approved for use in metastatic breast cancer in 2005 [31]. Via a process known as *nab*, the drug is non-covalently bound to albumin. The use of albumin as a DDS removes the need to include Cremophor, a solvent necessary in the conventional formulation and implicated in systemic toxicity and hypersensitivity reactions. In comparison to older formulations, such as Taxol, Abraxane has produced better response rates despite being used at a lower dose and demonstrated fewer systemic side effects, including decreased peripheral neuropathy and neutropenia. In addition to *nab*-paclitaxel, *nab*-docetaxel and *nab*-rapamycin are currently in early trials, demonstrating the success of albumin-based DDS [32,33]. A large number of other peptide or polymer-based nanoparticle DDS are also under investigation. The polymer SMANCS (polystyrene-co-maleic acid neocarcinostatin conjugate) has yielded some promising results in a number of solid tumors and is currently approved for clinical use in Japan for liver cancer [34]. Other polymers, including polylactide-co-glycolide (PLGA), polylactide (PLA), chitosan, and a class of lipid based carriers known as solid lipid nanoparticles (SLNs) have undergone investigation and demonstrated some promise, both *in vitro* and *in vivo* [35]. In addition to these, many other polymers are under investigation in various tumor models. As previously mentioned, these polymers are useful as DDS if they are readily metabolized by cellular processes to produce non-toxic byproducts, capable of incorporating lipid soluble active agents, and preferentially accumulate in target tissues either by the EPR effect or by incorporation of a molecule which actively targets the DDS to target cells.

### 2.4. Gold nanoshells

Gold and other heavy metals have recently drawn interest as a component of nanoparticle DDS known as nanoshells. Galvanic reactions, wherein a gold shell is “grown” around a silica core under suitable chemical conditions, can produce a molecule with a porous gold exterior and silica interior [36]. Manipulation of the silica core and the conditions of the reaction allow for the creation of variously sized and shaped shells, rods, or concentric spheres. Also, polymers such as PEG may be easily bound to gold by sulfur moieties to prevent uptake by RES and increase bioavailability of the nanoshell. Because nanoshells of various sizes and physical characteristics may be grown by altering the conditions of the reactions, DDS of the appropriate size to exploit the EPR principle may be easily created. Such molecules have demonstrated preferential accumulation within tumor cells *in vitro* and *in vivo* in animal models [37,38]. Further, the physical characteristics of the particle, chiefly its size and shape, influence its response to light. Particles may be tailored to either scatter light across a variety of wavelengths or to absorb certain wavelengths. Exposure of a nanoshell to the appropriate frequency of light causes coordinated excitation of electrons, which produces heat. This phenomenon is known as plasmonic resonance. The particular frequency which produces maximum plasmonic resonance is dependent on the physical properties of the nanoshell, which as previously noted, are easily manipulated. Plasmonic resonance of nanoshells subjected to near-infrared (NIR) light has generated sufficient heat to kill cancer cells in a murine model. These results raise the possibility that this technique may facilitate the visualization and local destruction of malignant cells under endoscopic examination in humans [39]. Utilization of nanoparticles tuned to frequencies of light with greater tissue penetration may allow similar therapy for less accessible tumors. Investigations into the use of nanoshells as contrast agents to enhance tumor imaging are underway and more creative uses are being explored. Such advances could produce previously unavailable early detection methods in tumors such as lung, pancreatic, and head and neck cancer and generate novel intervention modalities. Further, because cytotoxins and targeting molecules may be easily bound to nanoshells, they may further enhance the efficacy of more conventional therapy.

### 2.5. Heavy metal yolk-shells

When it enters cells, platinum induces DNA cross-linking, and ultimately, apoptosis. For this reason, it has long been used as a component of many chemotherapy regimens. The construction of platinum stabilizing nanoparticles was achieved in 2004 [40]. Over the past few years, multiple platinum containing platforms, termed “yolk-shell” particles, have demonstrated uptake into tumor cells and induction of apoptosis *in vitro* [41,42]. These particles employ a “shell” composed of a variety of compounds such as cobalt or silica, surrounding a hollow interior housing a heavy metal “yolk.” These shells are soluble in aqueous solutions which permit their dispersal in blood and other biologic media. Yin *et al* demonstrated uptake of these complexes by HeLa cells by endocytosis. After endocytosis, the outer shell is digested by intracellular enzymes, releasing platinum into the cytoplasm and inducing apoptosis by DNA cross-linking [43]. Iron based cores have also demonstrated cytotoxicity *in vitro* via generation of reactive oxygen species [44]. Because the shell remains intact until the DDS is actually inside the cell, this class of construct shields healthy tissues until endocytosis, ensuring targeted therapy. To further increase specificity, targeting molecules may be attached to the shell surface providing a means of active targeting. Like other heavy metal constructs, these particles have also drawn attention as targeted MRI contrast agents and as components of combination imaging and therapy [45]. Localized drug accumulation of shells containing magnetic components has been demonstrated via manipulation of magnetic fields [46]. It may be possible to guide magnetic nanoparticles to tumors via this technique as well, further improving localization of these particles to target tissues.

### 2.6. Carbon nanotubes

Carbon nanotubes are elongated carbon molecules with a spine formed by benzene rings The means of synthesizing carbon nanotubes was described by Iijima in 1991 [47]. Like other potential nanoparticle DDS, they may be easily tailored to utilize EPR by manipulation of their size. Their cylindrical shape also carries the advantage of a relatively high surface area to weight ratio, making them suitable for attachment of a large number of biologically active side chains and conjugates. They are also readily endocytosed *in vivo*, making them an attractive target for their use in chemotherapy [48,49]. Like other tailored drug delivery platforms, they are easily conjugated to a diverse array of molecules, facilitating their use as DDS, but because of their shape and high surface area to weight ratio, they may be able to deliver a higher dose of drug per particle than other DDS [50]. Studies of a paclitaxel-nanotube construct demonstrated superiority to Taxol via increased plasma half-life, increased tumor accumulation via EPR, and improved response to therapy in a murine breast cancer model [51]. A DDS based around nanotubes and doxorubicin has also demonstrated high levels of cytotoxicity in HeLa cells [52].

### 2.7. Peptide amphiphiles

In addition to providing novel DDS, advances in nanotechnology have also produced novel cytotoxins. Cytotoxic, or “killer,” peptides attack essential cell structures, such as cell membranes, inducing irreversible cellular damage, and ultimately, apoptosis. In the past, limited uptake into cells, clearance from the plasma by the RES, and degradation by proteases limited practical applications of these peptides. However, with the creation of a nanofiber DDS, these peptides may become another clinically useful cytotoxin. Nanofiber DDS self assemble via hydrostatic forces in a similar fashion to some other nanoparticle DDS via the incorporation of a hydrophobic “tail.” The active portion of the molecule is a beta pleated sheet designed to allow a large surface area for presentation of drug to cell surfaces. When equipped with a cytotoxic peptide, these structures have demonstrated cell uptake and killing *in vitro*. Recently, Standley *et al.* demonstrated cytotoxicity against cancer cell lines *in vitro* using a construct containing the KLAK peptide, which can interchalate into and disrupt plasma membranes [53]. Platforms completely composed of protein are appealing, as they could represent organic, completely biodegradable chemotherapeutics.

## 3. Active Targeting

While the EPR effect has been widely exploited in the delivery of various agents to tumor, there are clear limitations to passive targeting. Namely, healthy tissue is exposed to the effect of the drug and higher doses must be used to achieve the desired effect. Moreover, particles of the appropriate size can accumulate in other areas, such as skin capillary networks. These restrictions reduce the tolerable dose of chemotherapeutic agents and limit the effectiveness of therapy. Given the ease with which biologic molecules may be bound to tailored nanoplatforms, targeting molecules have been identified as the next improvement in drug delivery. In general, active targeting systems attempt to improve localization of the delivered drug to the tumor itself and facilitate uptake of the DDS by endocytosis.

### 3.1. Monoclonal antibodies

Monoclonal antibodies are generated via the immortalization of cloned plasma cells, producing a cell line which expresses an immunoglobulin specific for a single antigen. A wide variety of monoclonal antibodies are approved for clinical use in cancer with functions ranging from impairment of cell growth factors and cell signaling molecules and thus tumor growth to direct induction of apoptosis. A number of tumor associated antigens have been identified as targets and monoclonal antibodies targeting them are in clinical practice. The large number of previously developed monoclonal antibodies makes them promising ligands for use in novel nanoparticle based DDS. Examples include human epidermal growth factor receptor 1 (EGFR), human epidermal growth factor receptor 2 (her2), vascular endothelial growth factor (VEGF). These drugs may attack their targets by induction of the immune system via increased antigenicity or by direct interference with cell growth factors and receptors. Given their availability, a great deal of attention has been directed towards incorporating these molecules into nanoparticle based DDS, thus increasing specificity in drug delivery and promoting uptake by target cells. Some promising results have been generated in animal and human studies using doxorubicin conjugated with the BR96 antibody [54-56]. Her-2, CD20 and CD22 have also been used as ligands in the construction of actively targeted doxorubicin constructs [57-59]. Further, her-2 and CD19 have also been utilized in the construction of targeted liposomes containing vincristine and vinblastine [60,61].

### 3.2. Lectins

All cells express a diversity of signaling and attachment particles on the surface of the plasmalemma. The exact composition of these surface molecules is largely tissue-specific, and malignant tissue may similarly express a unique surface molecule profile. Lectins are one class of cell surface molecules which bind carbohydrate moieties. They are ubiquitous in cells and extremely diverse, making them favorable ligands for targeted delivery of nanoparticles. Cell specific lectins have been identified for a number of cell populations, leading to the development of lectin-drug complexes which have demonstrated cytoxicity in colorectal and liver cancer models [62-64]. Because lectins are such a diverse group of molecules, DDS targeted with lectins generate highly specific associations with their targets. The tissue specificity and diversity of this group of molecules suggest a promising future in targeted drug delivery.

### 3.3. Receptor analogues

Malignant cells are known to upregulate their expression of a large number of growth factors in order to facilitate their uncontrolled expansion. Some receptors which signal cell replication if activated are known to be an order of magnitude more common on the surface of malignant cells than healthy cells. Further, tumor cells often overexpress receptors designed for the uptake of essential growth factors, such as folate or iron. Because these molecules are expressed so much more frequently in malignant cells, they have become a target for drug delivery. Folate, the iron transport molecule transferrin, EGFR, *her-2*, and other receptors have all been identified as potential targets. Some of these targets have been exploited by monoclonal antibodies. Other receptors have been targeted via a variety of constructs. The transferrin receptor, for example, is thought to be expressed up to ten-times as frequently in some malignant cell lines as in healthy tissue [65]. Studies across a variety of tumors, including breast and colon adenocarcinoma, mesothelioma, and leukemia have demonstrated enhanced cytotoxicity in DDS targeted via the transferrin receptor [66-69]. Folate receptors are similarly overexpressed in malignant cells and several models have demonstrated efficacy in active targeting. Studies of folate receptor expression in squamous cell carcinoma of the head and neck have demonstrated a correlation between high expression of folate receptors and increased metastasis and poorer survival. A paclitaxel formulation incorporating folate termed HFT-T has demonstrated an improved response in a murine model of head and neck cancer [70,71].

## 4. Conclusions

A diverse array of nanoparticles are now under investigation, including a number of applications with promising effects (Table 1). The nanotechnology based DDS (Figure 1) produced to date have achieved some improvement in clinical practice over conventional therapy. With the increase in number of new particles being investigated and the increased interest in the field, oncology is poised to benefit from these applications. Nanoparticles have improved the bioavailability and safety of several commonly used chemotherapeutics. A number of these nanoparticle-based reformulations are currently available in clinical practice, while others are currently being tested in clinical trials. In the past, there has been greater reliance on the EPR effect to produce accumulation of drug in target tissues. These advances have reduced the toxicity and improved the drug delivery of several drugs, yet systemic side effects remain one of the largest barriers to effective therapy. The new actively targeted agents may further improve specificity in drug delivery and reduce side effects. However, delayed detection of numerous malignancies remains an important cause of mortality. Early detection and resection remains important for curing a large number of cancers. The widespread use of screening for some tumors, such as mammography, has improved outcomes and heightened the need for early detection in other tumors. Perhaps most promising of all is combination of novel DDS with diagnostic imaging to permit the fusion of diagnosis and therapy, allowing the simultaneous detection and treatment of malignancies in their early stage. Imaging in concert with therapy may further enhance the specificity of treatment via DDS activated by imaging. With the advent of such technologies, the future of therapy in oncology appears brighter.

## Figures and Tables

**Table 1.** Drug delivery systems (DDS) in clinical trials and practice.

**Name**	**Type of DDS**	**Type of Targeting**	**Stage of Approval for Clinical Practice**	**Therapeutic Agent**
**Doxil**	Liposome	Passive	Approved for Clinical Use	Doxorubicin
**DaunoXome**	Liposome	Passive	Approved for Clinical Use	Daunorubicin
**Abraxane**	Albumin-based polymer	Passive	Approved for Clinical Use	Paclitaxel
**Bexxar**	Immunoconjugate	Active	Approved for Clinical Use	Radioactive iodine
**SMANCS**	Nanopolymer	Passive	Approved for Clinical Use	Neocarzinostatin
**NK105**	Micelle	Passive	Phase 2	Paclitaxel
**Xyota**	Nanopolymer	Passive	Phase 3	Paclitaxel
**MBP-426**	Liposome	Active	Phase 1	Oxaliplatin
*References [72-76]*				
